# Advantage of straight walk instability in turning maneuver of multilegged locomotion: a robotics approach

**DOI:** 10.1038/srep30199

**Published:** 2016-07-22

**Authors:** Shinya Aoi, Takahiro Tanaka, Soichiro Fujiki, Tetsuro Funato, Kei Senda, Kazuo Tsuchiya

**Affiliations:** 1 Dept. of Aeronautics and Astronautics, Graduate School of Engineering, Kyoto University, Kyoto daigaku-Katsura, Nishikyo-ku, Kyoto 615-8540, Japan; 2 Dept. of Mechanical Engineering and Intelligent Systems, Graduate School of Informatics and Engineering, The University of Electro-Communications, 1-5-1 Choufugaoka, Choufu-shi, Tokyo 182-8585, Japan

## Abstract

Multilegged locomotion improves the mobility of terrestrial animals and artifacts. Using many legs has advantages, such as the ability to avoid falling and to tolerate leg malfunction. However, many intrinsic degrees of freedom make the motion planning and control difficult, and many contact legs can impede the maneuverability during locomotion. The underlying mechanism for generating agile locomotion using many legs remains unclear from biological and engineering viewpoints. The present study used a centipede-like multilegged robot composed of six body segments and twelve legs. The body segments are passively connected through yaw joints with torsional springs. The dynamic stability of the robot walking in a straight line changes through a supercritical Hopf bifurcation due to the body axis flexibility. We focused on a quick turning task of the robot and quantitatively investigated the relationship between stability and maneuverability in multilegged locomotion by using a simple control strategy. Our experimental results show that the straight walk instability does help the turning maneuver. We discuss the importance and relevance of our findings for biological systems and propose a design principle for a simple control scheme to create maneuverable locomotion of multilegged robots.

Legged locomotion is one of the versatile forms of mobility for terrestrial animals and artifacts. Locomotor behaviors differ in accordance with the number of legs. The use of many legs has advantages, such as the ability to avoid falling and to tolerate leg malfunction^1^,^2^,^3^,^4^. However, in the case of many legs, there are difficulties in motion planning and control due to the many intrinsic degrees of freedom and the dynamic interaction with diverse environments.

As an example, centipedes are arthropods that have a lot of legs. Although different species of centipedes have different numbers of legs, all have at least 15 pairs (some have 191 pairs) of legs as adults^5^,^6^. During centipede locomotion, many legs are in contact with the ground. The legs receive the reaction forces to support the body against gravity and produce propulsive and decelerating forces. This means that many legs must be physically constrained on the ground, and this constraint can impede their locomotion maneuverability. However, centipedes produce agile locomotion in diverse environments by using their many legs. The underlying mechanism for generating agile movements by using many legs remains unclear from biological and engineering viewpoints.

So far, to elucidate adaptive motor control in arthropods, biomechanical and physiological studies have measured the activities of their exoskeletal, muscular, and nervous systems during locomotion^7^,^8^,^9^,^10^,^11^,^12^,^13^,^14^,^15^,^16^. However, it is difficult to fully elucidate locomotion mechanisms solely from such measured data. To overcome the limitations of a single perspective, simple physical models^17^,^18^,^19^,^20^,^21^,^22^,^23^ and legged robots^24^,^25^,^26^,^27^,^28^,^29^,^30^,^31^ have recently been attracting attention. They can provide useful insight for biological systems and lead to a design principle for artifacts. In particular, to clarify the mechanism to create agile locomotion of cockroaches, Schmitt and Holmes^32^,^33^ developed a simple physical model composed of a planar rigid body and massless springy legs. Their model uses a control parameter for the root position of the legs to change the influence of the ground reaction force on the body dynamics. They showed that the stability of straight walking depends on the control parameter and the instability in straight walking helps the cockroach to make a quick turn during locomotion. Because the stability explains the capability to resist and recover from disturbances, performing a quick turn when the straight walk is unstable can change the walking direction more easily than performing the turn when the straight walk is stable. This means that straight walk instability contributes to turning maneuverability. Although their simplification of the physical model posed a limitation, the simulated locomotor behaviors captured the characteristics of straight walking and turning observed in cockroach locomotion. The results of this study, which suggested that cockroaches utilize the instability to improve turning mobility, provide a glimpse into motor intelligence in biological systems. However, centipedes have a much larger number of legs and a longer, more flexible body axis than cockroaches, and thus their turning strategies may differ.

In our previous work^34^, we developed a centipede-like multilegged robot composed of six body segments, each of which has a pair of legs. The body segments were passively connected through yaw joints with torsional springs. The robot experiments showed that the dynamic stability of a robot walking in a straight line changes through a supercritical Hopf bifurcation (a stationary solution becomes unstable and a new stable periodic solution appears at a critical value of a parameter) by changing the locomotion speed. The experiments also showed that the straight walk instability induces body undulations during locomotion. These findings were verified from a Floquet analysis using a simple physical model. Moreover, our robot captured the characteristics of centipede locomotion, such as the appearance of body undulations only during rapid locomotion and trends in the variation of body undulation amplitude and wavelength as a function of locomotion speed. These characteristics were evaluated by a comparison of the robot experimental results with the measured data of centipede locomotion^7^. In addition, our previous simulation study, which used a robot model and a simple physical model, showed that the straight walk stability also depends on the torsional spring constant^34^,^35^. That is, the body flexibility, which is a passive property in the body axis, changes the straight walk stability. Furthermore, the robot model simulation suggested that the stability influences the turning maneuverability. However, the physical condition of the simple model analysis and the robot model simulation was limited, and a part of the body-segment yaw joints was actively controlled during the turning task (i.e., not passively). The stability properties induced by the body axis flexibility and its contribution to the maneuverability were not clear.

In the present study, we improved our previous robot and control system to perform turning locomotion to demonstrate the relationship between the stability induced by the passive properties in the body axis and the maneuverability in multilegged locomotion in the real world. We first clarified the stability properties of a robot walking in a straight line in accordance with the body axis flexibility and then investigated how the stability properties influence the turning maneuverability by defining evaluation criteria to quantitatively investigate the relationship between stability and maneuverability. Our robot experimental results show that the body flexibility changes the stability of straight walking and that the straight walk instability does help the turning mobility, similar to the results of the modeling study of cockroaches. In this paper, we discuss the importance and relevance of our findings to understanding the underlying mechanisms in biological systems for manipulating the turning maneuverability and we propose a design principle for a simple control scheme to create maneuverable locomotion of multilegged robots.

## Results

### Robot

**Figure 1:**
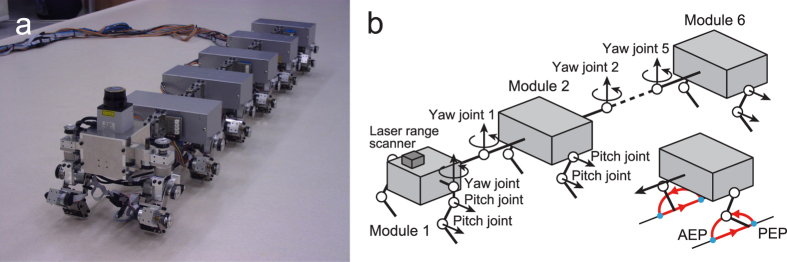
(**a**) Multilegged robot and (**b**) schematic model. The robot consists of six modules, each of which has one body segment and one pair of legs. Legs are controlled by two pitch joints, so that the leg tips follow a periodic trajectory including the anterior extreme position (AEP) and the posterior extreme position (PEP). Body segments are passively connected by yaw joints with installed torsional springs. The legs in the first module have additional yaw joints to change the walking direction. The laser range scanner is attached on the first module to find a position relative to a target.

We used a centipede-like multilegged robot (Fig. 1), which consists of six body segments and twelve legs. The total length is 135 cm. The body segments are connected by yaw joints (Yaw joints 1–5) installed with torsional springs (spring constant: *k*). Each leg has two pitch joints to walk and the legs in the first body segment have an additional yaw joint to control the walking direction by a laser range scanner. Although the body segments are passively connected, the leg joints are controlled by motors. The leg pitch joints are controlled so that the leg tips follow the desired movement composed of two parts: half of an elliptical curve, and a straight line (Fig. 1b). The straight line is parallel to the body segment. Therefore, when the leg yaw joint angles of the first module are fixed so that the leg tip trajectories are parallel to the body segment, the robot is expected to walk in a straight line while keeping the body segments parallel to each other. When the walking direction of the first body segment changes by manipulating the leg yaw joints, the body axis of the robot becomes curved due to the elasticity in the body-segment yaw joints. The elasticity allows the robot to change the walking direction.

### Instability in straight walking

Our previous study using a computer simulation of a robot model and a Floquet analysis with a simple physical model^34^,^35^ showed that when the spring constant *k* decreases under a threshold value, straight walking becomes unstable through a Hopf bifurcation, and body undulations appear. To clarify this bifurcation property in the real world, we performed robot experiments for a walk in a straight line by using varied spring constants for the torsional springs (*k* = 7.3, 8.7, 11, 15, 21, 41 and 450 Nmm/deg). We set all the body segments parallel to each other as the initial condition and the leg yaw joints in the first module were fixed.

**Figure 2:**
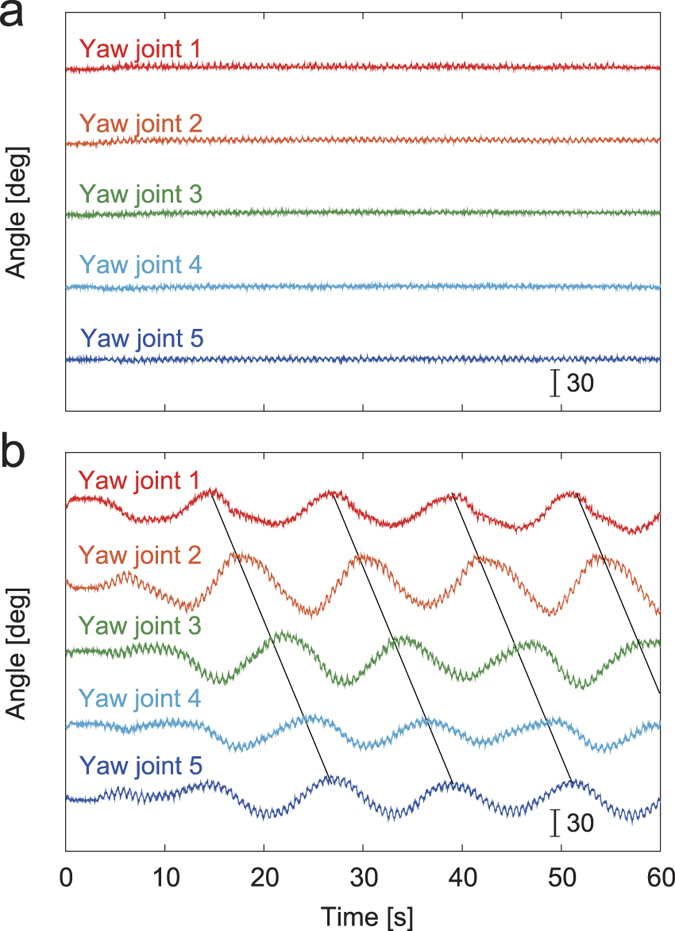
Appearance of body undulations under a threshold value of *k*. (**a**) Without appearance of body undulations for *k* = 450 Nmm/deg (see Supplementary movie S1) and (**b**) with appearance of body undulations for *k* = 7.3 Nmm/deg (see Supplementary movie S2).

**Figure 3:**
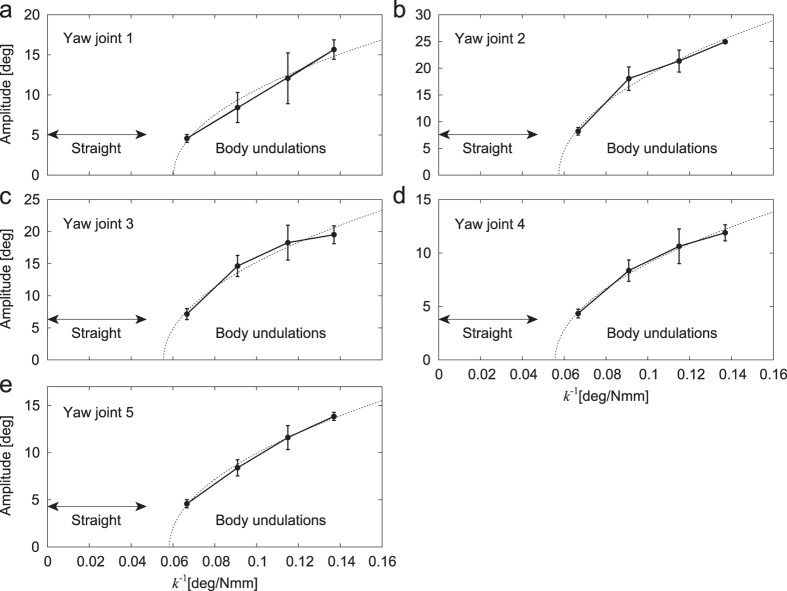
Amplitudes of body undulations in the body-segment yaw joints (**a**–**e**). The data points and error bars correspond to the means and standard errors, respectively, of the results of five experiments. Dotted lines are fitted functions using the square root of *k*^−1^ to evaluate the bifurcation point.

When we used large spring constants for torsional springs in the body-segment yaw joints, the robot kept walking in a straight line as expected, and the body segments were aligned without producing body undulations (Fig. 2a, Supplementary movie S1). However, when the spring constant decreased under a threshold value, body undulations appeared (Fig. 2b, Supplementary movie S2). More specifically, body undulations occurred for *k* = 7.3, 8.7, 11, and 15 and not for *k* = 21, 41, and 450 Nmm/deg. Figure 3 shows the amplitudes of body undulations in each body-segment yaw joint for *k*^−1^ calculated by the Fourier transform. The data points and error bars correspond to the means and standard errors, respectively, of the results of five experiments. These amplitudes vary with *k*^−1^ and show the Hopf bifurcation. These results were fitted by the square root of *k*^−1^ to evaluate the bifurcation point 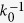
36, which gives 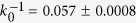 (SE) from five body-segment yaw joints.

### Turning maneuverability

**Figure 4:**
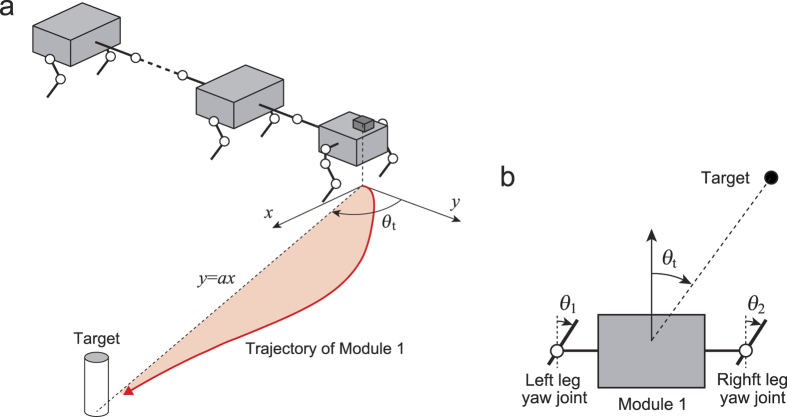
(**a**) Turning task to approach a target on the floor (*x-y* plane). *θ*_*t*_ is the relative angle between the first module and the target. *y* = *ax* indicates the line from the initial position to the target. The colored area enclosed by the first module trajectory and the line (*y* = *ax*) shows one evaluation criterion of the turning performance. (**b**) Turning control by the leg yaw joints *θ*_1_ and *θ*_2_ of the first module based on *θ*_*t*_.

To investigate the relationship between the revealed stability properties in a straight walk and the turning mobility, we performed robot experiments for a quick turn to approach a target located on the floor (*x*-*y* plane). The leg yaw joints *θ*_1_ and *θ*_2_ of the first module were controlled based on the relative angle *θ*_*t*_ between the first module and the target monitored by the laser range scanner (Fig. 4). We used *θ*_*t*_ = 80° with a 4 m distance from the first module to the target and set all body segments parallel to each other as the initial condition. This experiment was designed so that the first module determined the walking direction and the other modules followed the first module through passive connections of the body-segment yaw joints to achieve the turning task.

**Figure 5:**
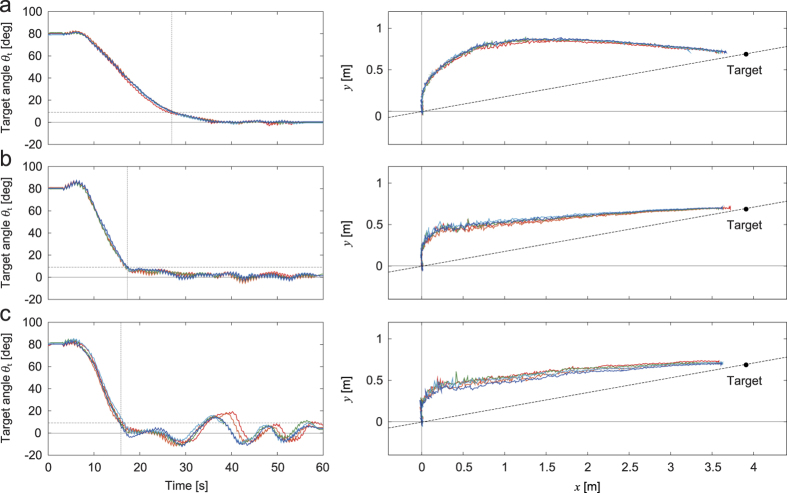
Relative target angle *θ*_*t*_ and trajectory of the first module *f*(*x*) during the turning task for (**a**) *k*^−1^ = 0.0022 (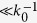) (see Supplementary movie S3), (**b**) *k*^−1^ = 0.048 (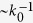) (see Supplementary movie S4), and (**c**) *k*^−1^ = 0.115 (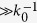) (see Supplementary movie S5). Five experimental results are shown in each figure.

Figure 5 shows the time profile of the relative target angle *θ*_*t*_ and the trajectory of the first module on the floor during the turning task, where **a**, **b** and **c** show the results for a large spring constant (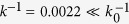), for a spring constant close to the bifurcation point (
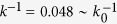
) and for a small spring constant (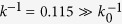), respectively (Supplementary movies S3,4–5). When the spring constant was close to the bifurcation point (Fig. 5b), the robot was quickly oriented to the target and the deviation of the first module trajectory on the floor from the line between the initial position and the target (dotted lines) was small. In contrast, for the large spring constant (Fig. 5a), it took much time to change the walking direction and the first module trajectory bulged outward. The bulged shape indicates a large overshoot to approach the target. When the spring constant was small (Fig. 5c), although the robot was quickly oriented to the target direction, the relative target angle fluctuated greatly during the task. In addition, when we used *k* = 7.3 Nmm/deg (*k*^−1^ = 0.137), which was the smallest spring constant in our experiments, neighboring body segments easily clashed and our robot could not achieve the turning task well.

**Figure 6:**
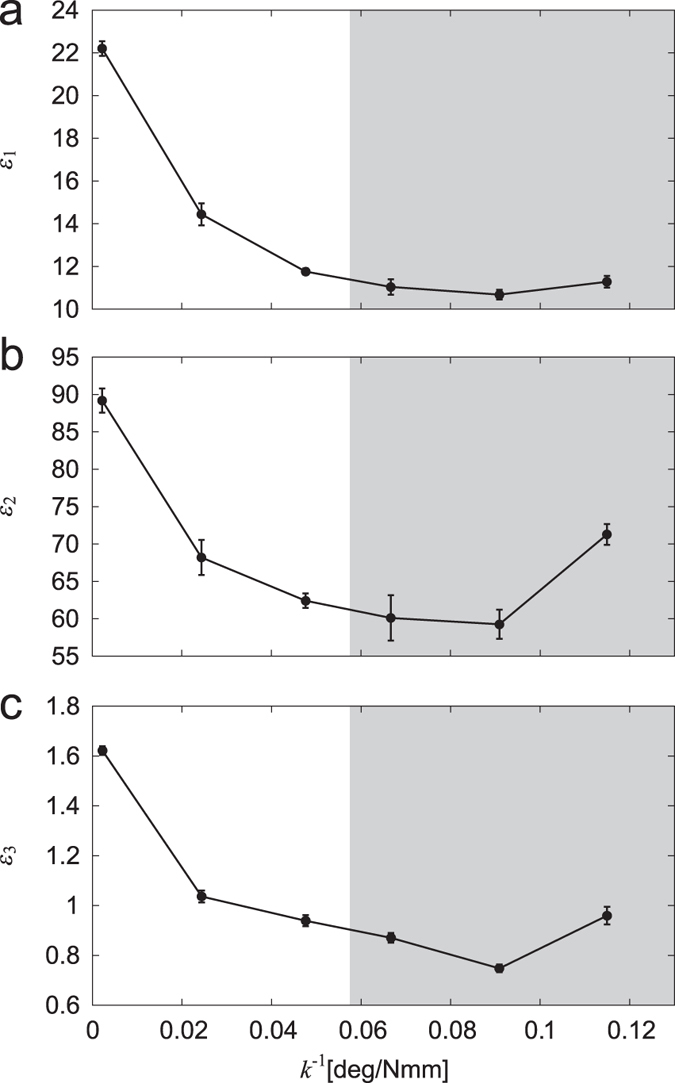
Evaluation criteria (**a**) *ε*_1_, (**b**) *ε*_2_, and (**c**) *ε*_3_ of the turning task for *k*^−1^. Gray regions indicate the estimated unstable region of the straight walk. The data points and error bars correspond to the means and standard errors, respectively, of the results of five experiments.

To quantitatively clarify the turning performance in accordance with the spring constant, we employed three evaluation criteria, *ε*_1_, *ε*_2_, and *ε*_3_. Figure 6 shows the results for *k*^−1^, where the data points and error bars correspond to the means and standard errors, respectively, of the results of five experiments, and the gray regions indicate that the straight walk is unstable, as estimated from Fig. 3. The first criterion *ε*_1_ shows the time that our robot took to reduce the relative target angle to less than 10% of the initial value; the time is used to evaluate how quickly our robot changes the walking direction to point to the target. The second criterion *ε*_2_ is the integration of the absolute value of the relative target angle during the turning task; the integration is used to estimate how quickly and stably our robot is oriented to the target. The third criterion *ε*_3_ indicates the deviation of the first module trajectory on the floor from the line between the initial position and the target (*y* = *ax*, *a* = tan10°); the deviation is used to evaluate how small the overshoot of the robot trajectory is to approach the target. For these criteria, smaller values indicate better turning performance. As shown in the figures, when the spring constant is large so that walking in a straight line is stable, these criteria have large values. Although these criteria decrease as the spring constant decreases, they show an increase, especially *ε*_2_ and *ε*_3_, at *k*^−1^ = 0.115. This means that the turning performance increases as the straight walk becomes unstable, but that a large instability degrades the performance.

## Discussion

As should be clear by now, stability and maneuverability are important factors for evaluating locomotor performance. The stability is related to the capability in the moving direction to resist and recover from disturbances. In contrast, the maneuverability is related to the ability to voluntarily change the direction. These properties have a strong connection in locomotion. The connection prominently appears in locomotion generated through the dynamic interactions of aerodynamics and hydrodynamics. For example, some aquatic animals and aircrafts utilize instability to enhance their maneuverability. Sea lions have relatively large flippers and highly flexible bodies, and execute turning by body flexion and using their flippers. Their flippers are located near the center of gravity and their morphology promotes rotational and translational instabilities, which enhances turning performance^37^. Fighter aircrafts use a high angle of attack and a high roll rate, which cause the loss of stability, to achieve high maneuverability^38^. In particular, the F-16 was designed to be aerodynamically unstable to enhance maneuverability^39^.

Legged locomotion is characterized by the dynamic interactions between the feet and the contact surface and by the intermittency of the interactions due to the discrete events of foot contact and lift-off in periodic leg movement. Mammals with erect legs have a high center of mass and use body leaning to enhance their turning maneuverability^33^,^40^. In contrast, arthropods with sprawling legs have a low center of mass and thus have difficulty in using the effect of leaning. Their locomotor behaviors are limited to being almost planar in a horizontal plane and the stability characteristics of the walking direction in the plane are assumed to be more crucial for the turning mobility as compared with mammals. However, the relationship between stability and maneuverability in arthropod locomotion is not well understood, partly because it is difficult to quantify stability and maneuverability in their locomotion^41^. Physical modeling and robotics studies are useful to overcome such a difficulty^32^,^33^. In the present study, we quantitatively evaluated the stability based on the appearance of body undulations (Fig. 3), which indicates a Hopf bifurcation, and the maneuverability by focusing on a quick turning task and defining three precise criteria (Fig. 6). These criteria suggest an important relationship between stability and maneuverability in multilegged locomotion.

In centipede locomotion, body undulations are absent at slow speeds and appear at faster speeds^13^,^14^. It has been suggested that rapid stepping movements induce body undulations and such undulations impede centipede motion and are resisted by the muscles along the body axis. The torsional springs in the body-segment yaw joints of our robot are based on this suggestion^34^ and determine the body axis flexibility. However, a conflicting study reported that the body axis muscles support body undulations^7^, and so the control mechanisms of the body axis movements remain questionable. In aquatic animals, the body flexibility plays an important role in locomotion^42^ and contributes to the turning performance^43^,^44^. In legged locomotion, changing the leg movement relative to the body influences the stability and maneuverability, as suggested in the case of hexapods^32^,^33^,^41^. Meanwhile, for arthropods that have a long body axis relative to the legs, such as centipedes, the body flexibility is another possibility. In the present study, we assumed that the legs of the first module create the driving force to change the walking direction and clearly determine the contribution of the body axis flexibility to stability and maneuverability.

Although our robot has only six pairs of legs, adult centipedes have at least 15 pairs of legs (some have 191 pairs of legs)^5^,^6^. Our previous works^34^,^35^ investigated the influence of the number of legs by using a robot model and a simple physical model, and clarified that three or more pairs of legs show a Hopf bifurcation. Although the bifurcation value of the body axis flexibility depends on the number of legs, the bifurcation does occur irrespective of the number of legs. In addition to the locomotion speed and the number of legs, physical and environmental conditions, such as the body weight and length and the floor friction coefficient, affect the locomotion performance. Our previous studies also investigated the influence of such conditions and showed that although the bifurcation value depends on the conditions, the appearance of the bifurcation does not depend on the conditions. In particular, floor friction is difficult to model due to the complexity of the physical interaction between the feet and the contact surface. The simulation studies used various well-known friction models, such as the linear damper model, the spring-damper model, and the Coulomb friction model, and showed that the bifurcation occurs regardless of such models.

Even though the instability helps the turning maneuver, stability and maneuverability are generally a trade-off^45^,^46^,^47^,^48^. Strong instability impedes locomotor performance, as observed in our turning experiments (Fig. 6). It is important to have an appropriate relationship between stability and maneuverability to produce the required motor functions for locomotion and to control the relationship according to the situation. Our robot experiments show that the body axis flexibility controls the stability (Fig. 3) and also the maneuverability (Fig. 6). Tuning the body axis flexibility, e.g., by appropriately timed muscle activation as seen in aquatic animals^42^,^49^, would absolutely be useful.

In the present study, the stability changed through a bifurcation via the body axis flexibility. Bifurcation plays an important role in various motor behaviors in terrestrial animals, such as gait transition^50^,^51^,^52^,^53^ and body sway in quiet standing^54^,^55^. Although it is difficult to fully clarify if and how much multilegged arthropods use dynamic instability for turning behaviors, our robot experimental results give a possible mechanism and provide meaningful insight for biological sciences.

For the control design of the locomotion of artifacts, maneuverability is an important factor. In particular, biologically inspired robots that use the body axis for propulsion in locomotion, such as snake and fish robots, have achieved maneuverable locomotion comparable to that of animals^47^,^56^,^57^,^58^,^59^. However, legged robots still have difficulties in producing high maneuverability in their locomotion. This is partly because they need control to avoid falling down in addition to control of the walking direction. The intermittency of the interaction with the contact surface is inevitable for legged locomotion and makes the avoidance of falling difficult. So far, the main purpose of the control design of legged robots has focused on the avoidance of falling through criteria based on, for example, a supporting polygon and a zero moment point (ZMP)^60^. Maneuverability has not been well investigated. However, as the number of legs increases, it becomes easier to avoid falling, as seen in centipedes. Instead, as the number of legs increases, the degrees of freedom to be controlled increase, and both motion planning (e.g., where the feet contact) and control become more difficult. Furthermore, the number of contact legs also increases, and this may impede maneuverable locomotion. Until now, no design principle has been proposed for the control of robots with many legs to create maneuverable locomotion.

Even when all robot movements are planned in real time through huge computations based on the robot model, and when tasks and planned motions are generated, robust locomotion against uncertainties, such as modeling errors and environmental variations, is not necessarily produced. Locomotor performance degrades as the robot and the environment become more complex. Designing a simple control system based on fundamental dynamical principles is more useful for legged robots than a control system based on such precise motion planning and control. For example, biped robots based on passive dynamic walking^61^ (walking down a shallow slope without any actuators or controllers) produced efficient locomotion due to inherent body dynamics and simple controllers^62^. The hexapod robot RHex series was designed to have self-stabilization properties, based on the spring-loaded inverted pendulum (SLIP) model, embedded in their body dynamics, and they successfully produced agile and robust locomotion in various environments^27^,^28^,^63^,^64^. Our robot used a simple control strategy, where the legs in the first module determine the walking direction and turning is achieved through the passive dynamics in the body axis.

In general, the design of the controller of artifacts focuses on the stabilization of the systems. However, our robot experiments show that the active use of instability leads to improvement of locomotor functions. Such instability is useful, as shown for artifacts such as aircraft^38^,^39^. However, high instability impairs the locomotor performance, and thus it is important to control the stability in accordance with the required functions. In the future, we will investigate both the optimal distribution of the spring constant in the body-segment yaw joints and adaptive tuning to enhance the maneuverability of the robot.

## Methods

### Multilegged robot

Our multilegged robot consists of six body segment modules (Modules 1–6), as shown in Fig. 1. Each module is composed of a single body and one pair of legs. Each leg has two links connected by pitch joints, while that in the first module (Module 1) has an additional link connected by a yaw joint to control the walking direction. Each leg joint is manipulated by an encoder-equipped motor. The body segments are passively connected by yaw joints (Yaw joints 1–5) installed with torsional springs and potentiometers. We used the same spring constant for the yaw joints and compared seven spring constants (*k* = 7.3, 8.7, 11, 15, 21, 41 and 450 Nmm/deg) to investigate the dependence of walking performance on the straight walk stability. Module 1 has a laser range scanner (Hokuyo, URG-04LX) to find the relative position of a target for turning. Table 1 shows the physical parameters of the robot.

The robot was controlled by an external host computer (Intel Pentium 4 2.8 GHz, RT-Linux) with 2 ms intervals, and both computer control and electric power were provided via external cables. During the experiments, the computer control and electric power cables were kept slack and suspended to avoid influencing the robot’s locomotor behavior. The robot walked on a wooden flat floor with a vinyl floor mat to suppress slipping.

### Locomotion control system and experiments

#### Leg control for straight walking

To produce straight walking of the robot, we controlled the leg movement by using the two pitch joints in each leg to follow the desired movement, which consists of two parts: half of an elliptical curve that starts from the posterior extreme position (PEP) and ends at the anterior extreme position (AEP), and a straight line from the AEP to the PEP (Fig. 1b). In the straight line section, the leg tips moved from the AEP to the PEP in the opposite walking direction at a constant speed parallel to the body. We used 0.29 s for the duration of the half elliptical curve, 0.31 s for the duration of the straight line and 3 cm for the distance between the AEP and he PEP on each leg. The contralateral legs in each module were manipulated to move in antiphase, and the relative phase between the ipsilateral legs on adjacent modules was set to 2*π*/3 rad. When the leg yaw joint angles of Module 1 were fixed so that the leg tip trajectories were parallel to the body segment, our robot was expected to walk in a straight line while keeping the body segments parallel to each other, because torsional springs were installed on the body-segment yaw joints and the leg tips moved parallel to the body segments at an identical speed for all legs.

#### Turning control and performance evaluation

For a quick turn task of our robot, we made the robot approach a target located on the floor in a largely different direction from where the robot was oriented (Fig. 4a). For that purpose, we used the relative angle *θ*_*t*_ of the first module measured by the laser range scanner and the leg yaw joints *θ*_1_ and *θ*_2_ of the first module (Fig. 4b). Specifically, we determined the desired angles 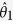 and 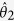 of *θ*_1_ and *θ*_2_ for each gait cycle (
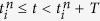
, 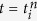 is the time when the desired leg tip is at the PEP for the *n*th gait cycle, and *T* is the gait cycle duration (=0.6 s)) by


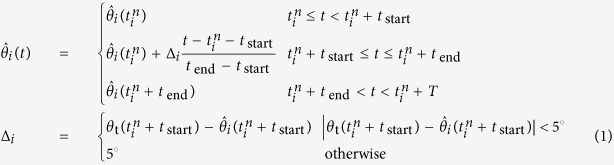


where *t*_start_ and *t*_end_ were set to 40% and 80%, respectively, of the duration of the half elliptical curve of the leg tip trajectory (=0.12 and 0.23 s). This means that each leg changed its yaw direction to the target only during the swing phase with 5° of the maximum turning angle for one gait cycle. The robot iterated this procedure in the legs of the first module for the gait cycles (after *t* = 5 s), and the turning task was complete when *θ*_*t*_ converged to 0. We performed robot experiments of this turning task for various spring constants of the torsional springs to investigate the dependence of the turning mobility on spring constant *k*. We used *θ*_*t*_ = 80° and 4 m for the distance between the first module and target and set all body-segment yaw joint angles to zero as the initial condition.

To quantitatively investigate the turning performance of our robot, we defined three evaluation criteria, *ε*_1_, *ε*_2_, and *ε*_3_. For the first criterion, *ε*_1_, we examined the time needed for our robot to reduce the relative angle *θ*_*t*_ to less than 10% of the initial value in order to evaluate how quickly the robot changed the walking direction to point to the target. We defined the second criterion *ε*_2_ by


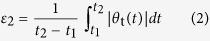


where *t*_1_ = 5 s (start time of turning) and *t*_1_ = 55 s (*θ*_*t*_ sufficiently converges to 0 for stable cases and shows some oscillations for unstable cases by this time). This criterion estimated how quickly and stably our robot was oriented to the target. For the third criterion, *ε*_3_, we used


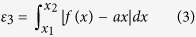


where *y* = *f*(*x*) is the trajectory of the first module on the floor during the turning task, *x* = *y* = 0 is the initial position, *y* = *ax* is the line connected from the initial position to the target (*a* = tan10°), *x*_1_ = *x*(*t*_1_), and *x*_2_ = *x*(*t*_2_). The position of the first module on the floor was calculated by measuring not only the target but also other landmarks located on the floor by using the laser range scanner. The third criterion calculated the area enclosed by the trajectory of the first module (*y* = *f*(*x*)) and the line (*y* = *ax*) to evaluate how much the first module deviated from the line (*y* = *ax*) and how small the overshoot was to approach the target (Fig. 4a).

## Additional Information

**How to cite this article**: Aoi, S. *et al*. Advantage of straight walk instability in turning maneuver of multilegged locomotion: a robotics approach. *Sci. Rep.* **6**, 30199; doi: 10.1038/srep30199 (2016).

## Supplementary Material

Supplementary Information

Supplementary Movie S1

Supplementary Movie S2

Supplementary Movie S3

Supplementary Movie S4

Supplementary Movie S5

## Figures and Tables

**Table 1:** Physical parameters of the multilegged robot.

Module 1			Module 2–6		
Link	Parameter	Value	Link	Parameter	Value
Body	Mass [kg]	1	Body	Mass [kg]	0.68
	Length [cm]	10		Length [cm]	10
	Width [cm]	20		Width [cm]	20
Upper Leg	Mass [kg]	0.26	Upper Leg	Mass [kg]	0.25
	Length [cm]	3.2		Length [cm]	5
Middle Leg	Mass [kg]	0.25	Lower Leg	Mass [kg]	0.03
	Length [cm]	5		Length [cm]	5
Lower Leg	Mass [kg]	0.03			
	Length [cm]	5			
