# Supplementary material for: Advantage of straight walk instability in turning maneuver of multilegged locomotion: a robotics approach
Source: Sci Rep. 2016 Jul 22;6:30199. doi: 10.1038/srep30199 (PMC4957114; doi:10.1038/srep30199)
Supplement: Supplementary Information [file srep30199-s1.pdf]

Supplementary Information:
Advantage of straight walk instability in turning maneuver of multilegged locomotion: a robotics approach

Shinya Aoi[1,*], Takahiro Tanaka[1], Soichiro Fujiki[1], Tetsuro Funato[2], Kei Senda[1], and Kazuo Tsuchiya[1]

[1]Dept. of Aeronautics and Astronautics, Graduate School of Engineering, Kyoto University, Kyoto daigaku-Katsura, Nishikyo-ku, Kyoto 615-8540, Japan
[2]Dept. of Mechanical Engineering and Intelligent Systems, Graduate School of Informatics and Engineering, The University of Electro-Communications, 1-5-1 Choufugaoka, Choufu-shi, Tokyo 182-8585, Japan
[*]shinya_aoi@kuaero.kyoto-u.ac.jp

Supplementary movies

We prepared five supplementary movies to show the stability and turning performance of the robot:

S1. Walking in a straight line with a large spring constant for the torsional springs in the body-segment yaw joints.

S2. Appearance of body undulations by using a small spring constant for the torsional springs in the body-segment yaw joints.

S3. Quick turning by using a large spring constant relative to the bifurcation point for the body-segment yaw joints.

S4. Quick turning by using a spring constant close to the bifurcation point for the body-segment yaw joints.

S5. Quick turning by using a small spring constant relative to the bifurcation point for the body-segment yaw joints.
